# HYGRIP: Full-Stack Characterization of Neurobehavioral Signals (fNIRS, EEG, EMG, Force, and Breathing) During a Bimanual Grip Force Control Task

**DOI:** 10.3389/fnins.2020.00919

**Published:** 2020-10-26

**Authors:** Pablo Ortega, Tong Zhao, A. Aldo Faisal

**Affiliations:** ^1^Brain & Behavior Lab, Department of Computing and Department of Bioengineering, Imperial College London, London, United Kingdom; ^2^EPSRC Centre for High Performance Embedded and Distributed Systems, Imperial College London, London, United Kingdom; ^3^UKRI Centre in Artificial Intelligence for Healthcare, Imperial College London, London, United Kingdom; ^4^Data Science Institute, London, United Kingdom

**Keywords:** electroencephalography, near-infrared spectroscopy, power-grip, non-invasive, brain-computer interface, sensor-fusion, data set, continuous decoding

Brain-computer interfaces (BCIs) have achieved important milestones in recent years, but the majority of breakthroughs in the continuous control of movement have focused on invasive neural interfaces with motor cortex or peripheral nerves. In contrast, non-invasive BCIs have primarily made progress in continuous decoding using event-related data, while the direct decoding of movement command or muscle force from brain data is an open challenge. Multi-modal signals from human cortex, obtained from mobile brain imaging that combines oxygenation and electrical neuronal signals, do not yet exploit their full potential due to the lack of computational techniques able to fuse and decode these hybrid measurements. To stimulate the research community and machine learning techniques closer to the state-of-the-art in artificial intelligence, we release herewith a holistic data set of hybrid non-invasive measures for continuous force decoding: the Hybrid Dynamic Grip (HYGRIP) data set. We aim to provide a complete data set that comprises the target force for the left/right-hand cortical brain signals in form of electroencephalography (EEG) with high temporal resolution and functional near-infrared spectroscopy (fNIRS), which captures in higher spatial resolution a BOLD-like cortical brain response, as well as the muscle activity (EMG) of the grip muscles, the force generated at the grip sensor (force), and confounding noise sources, such as breathing and eye movement activity during the task. In total, 14 right-handed subjects performed a uni-manual dynamic grip force task within 25–50% of each hand's maximum voluntary contraction. HYGRIP is intended as a benchmark with two open challenges and research questions for grip-force decoding. The first is the exploitation and fusion of data from brain signals spanning very different timescales, as EEG changes about three orders of magnitude faster than fNIRS. The second is the decoding of whole-brain signals associated with the use of each hand and the extent to which models share features for each hand or, conversely, are different for each hand. Our companion code makes the exploitation of the data readily available and accessible to researchers in the BCI, neurophysiology, and machine learning communities. HYGRIP can thus serve as a test bed for the development of BCI decoding algorithms and responses fusing multimodal brain signals. The resulting methods will help understand limitations and opportunities to benefit people in health and indirectly inform similar methods, answering the particular needs of people in disease.

## Introduction

Brain-computer interfaces (BCIs) offer communication pathways for people with motor disorders to regain agency in their body and environment (Wolpaw et al., 2002). Since their first demonstration almost 50 years ago (Vidal, 1973, 1977; Wolpaw et al., 2000), BCIs have undergone a steady evolution. Invasive BCIs have achieved significant milestones in continuous signal read out from nervous system activity such as speech decoding (Guenther et al., 2009; Bocquelet et al., 2016; Anumanchipalli et al., 2019), robotic or own arm continuous control (Pfurtscheller et al., 2003; Hochberg et al., 2012), and even grip control with touch sense recovery (Ganzer et al., 2020). Non-invasive BCIs have also succeeded in the continuous control of trajectories after users learned to modulate event-related desynchronization (ERD) (Wolpaw and McFarland, 2004; Royer et al., 2010; Meng et al., 2016). However, while force is central to motor control (Westling and Johansson, 1984; Ostry and Feldman, 2003), its continuous non-invasive decoding is still challenging even in the offline case, and only modest accuracies have been reported using electroencephalography (EEG) (Paek et al., 2019). Previous attempts at decoding force from non-invasive measures have focused on the classification of discrete force variables using EEG (Jochumsen et al., 2013; Wang et al., 2017). In the hybrid case of recording cortical brain signals non-invasively, by combining EEG and functional near-infrared spectroscopy (fNIRS), Yin et al. (2015) showed that the combination of both measures increased the classification accuracies of different forces featured during imagined hand clenching by 1–5% compared to EEG or fNIRS alone. However, the lack of methods successfully integrating both measures in continuous decoding is still limiting the benefits of hybrid setups (Ahn and Jun, 2017).

We have shown that combining both multi-modal BCI (e.g., Thomik et al., 2013; Belić and Faisal, 2015; Xiloyannis et al., 2017) and the use of state-of-the-art machine learning—from introducing Deep Learning for EEG-BCI in 2015 (Walker et al., 2015) to data-efficient methods for BCI decoding that minimize the need for collecting data from individual end users (Xiloyannis et al., 2017; Ortega et al., 2018)—can help BCI research if data is collected with a machine learning use in mind. To stimulate the development of advanced multi-modal BCI techniques we present the Hybrid Dynamic Grip (HYGRIP) data set^1^. HYGRIP includes hybrid non-invasive and co-located brain activity measures as well as the hand contraction and muscular electrical behavioral activities during a hand-grip task with fast dynamics. The companion repository^2^ digests the raw data into a format that makes it at a data readiness level suitable for immediate use by machine learning engineers (Lawrence, 2017) without having to go through a lengthy process of cleanup and reshaping of the data, which we believe will facilitate drawing in more data science and machine learning experts to the exciting problem of BCI.

## Participants

Fourteen (*N* = 14, anonymized IDs from A to N) healthy, right-handed volunteers participated in the production of this data set. Handedness was confirmed by the Edinburgh inventory (Oldfield, 1971) for all participants. None reported a history of neurological, cardio-respiratory, or physical disorders. Imperial College Research Ethics Committee approved all procedures, and all participants gave their written informed consent. The experiment complied with the Declaration of Helsinki for human experimentation and national and applicable international data protection rules.

## Motor Control Task

The motor task consists of a left/right-hand grip, each hand being a different condition in the experiment. The task consisted of 10 consecutive contraction (1.55 s)/relaxation (0.55 s) periods that introduced rapid changes of force. Subjects were instructed and received visual feedback to exert forces in the 25–50% of their maximum voluntary contraction (MVC) following the pace of the 1.55 s contraction/0.55 s relaxation periods. The 25–50% MVC target range acted as a soft margin within which the subjects had to produce a contraction rather than a varying force they had to track. The MVC target range was implemented in this way to reduce the effect of visual feedback during the task that was provided through a computer screen for contractions out of the task range.

Due to the velocity of the contraction/relaxation periods, we do not consider each period a single trial but the consecutive 10 periods as a single trial of the task emphasizing the velocity of the execution. Note also that the much slower fNIRS signals need longer times to show a response, and using each single period independently of previous ones could hinder the resolution of the response. Participants were also instructed to prioritize the gripping pace rather than accurately matching the visual cues since the latter was too demanding for the levels of contraction required. Details follow.

**Figure 1:**
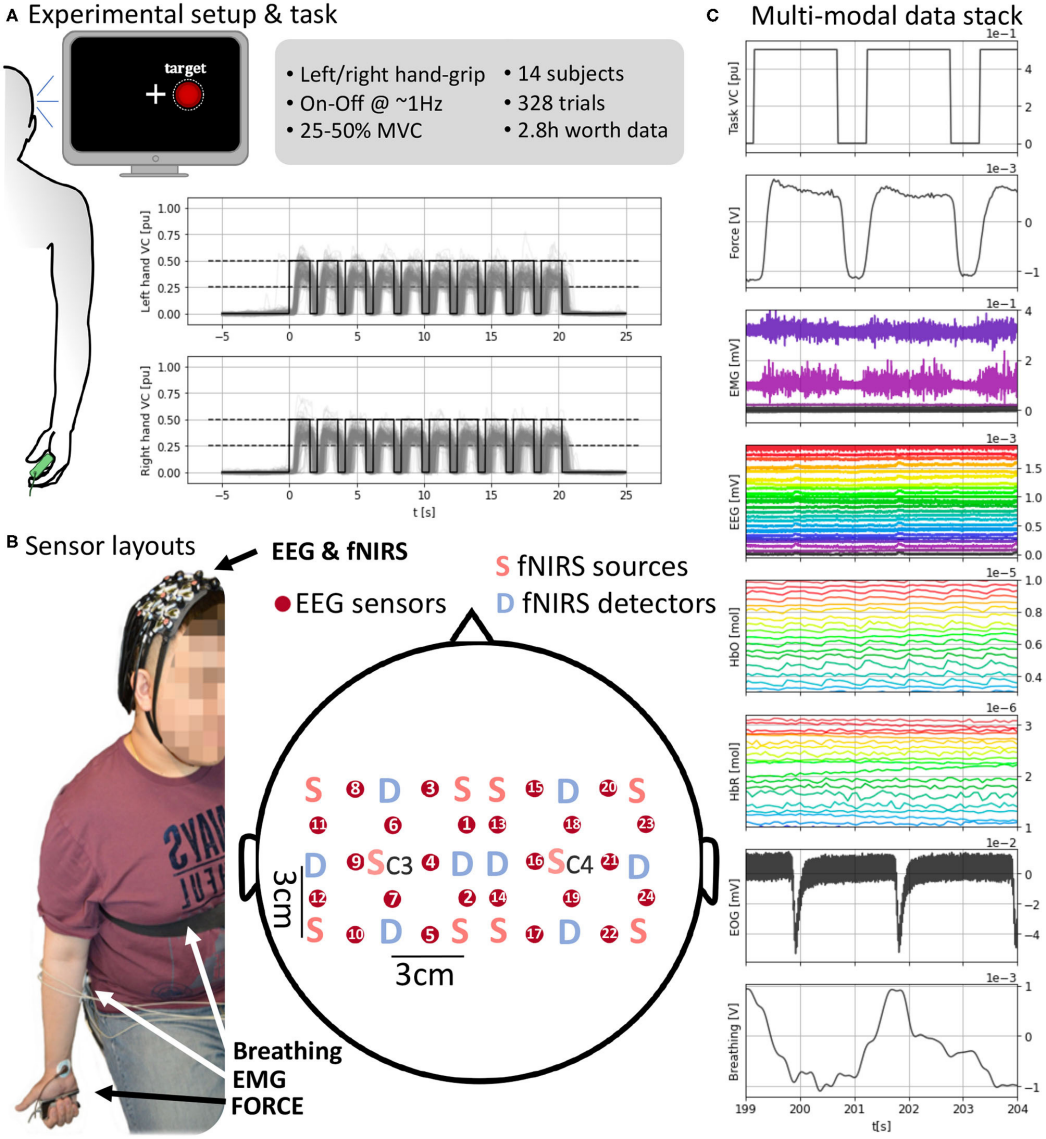
**(A)** (Left) Experimental setup with visual feedback. (Top right) Task and data set details. (Bottom right) Task execution across subjects time-locked to the “Go” cue at *t* = 0 s. **(B)** Sensor layout and EEG and fNIRS sensor arrangement. fNIRS light sources and detectors are placed in a 5 × 5 symmetrical grid leaving a required 3 cm distance. EEG electrodes, red circles, are placed between each pair of sources and detectors overlaying the fNIRS sensing area so fNIRS and EEG measures are co-located. Each grid is centered around C3 and C4 for the respective hemisphere. **(C)** A subject's full stack of neurobehavioral data (5 s, selected channels per modality for readability). (Top-bottom) Force target for the right hand; force produced by subject's right hand; EMG channels from right forearm; EEG channels; HbO (oxyhemoglobin); HbR (deoxygenated hemoglobin); EOG with two eye blinks; breathing from chest strap stretch sensor. Units in the bottom x-axis and each corresponding y-axis.

Subjects sat in front of the computer screen with their arms relaxed and ergonomically hanging down, i.e., the arms were naturally straight downwards while holding the force transducer (Figures 1A,B). Subjects were instructed to keep this relaxed posture and reminded to maintain it throughout the experiment.

At the start of the experiment, subjects were asked to produce their maximum voluntary contraction (MVC) with each hand. The MVC was used to calibrate the feedback for each hand independently. To obtain reliable MVC estimates, MVC grips were repeated 10 times for 1 s with each hand and following paced auditory cues. The MVC was computed as the average of the maximum force across the 10 trials. For the experimental task, participants had to generate unimanual hand-grip contractions in the 25–50% MVC range during the 21 s gripping period. The contraction was computed as the ratio Force/MVC. The hand used for the grip varied randomly between trials with equal probability. The force transducer was handed by the experimenter to the subject within their immediate reach before each trial and removed after trial completion.

A computer screen in front of the participants delivered the feedback at an approximate 60 Hz refreshing rate (Figure 1B). A cross indicating the center of the screen was used as fixation point during the experiment and subjects were instructed to stare at it to avoid eye movements. A white discontinuous circumference was used as a visual target and appeared at either side of the cross ipsilateral to the hand to be used in each trial. This visual target matched the 25–50% of the maximum voluntary contraction (MVC) of the subject for that hand and paced the contraction-relaxation periods. When the visual target appeared (shown for 1.55 s) the instruction was to contract and, when it disappeared (not shown for 0.55 s), to relax. A red filled circle indicated the real-time contraction level and subjects aimed to fill the discontinuous target circle with red. Visual feedback was only coupled to grips outside the desired range of contraction (25–50% MVC) making the red circle bigger or smaller than the target. Otherwise, the red circled filled the white discontinuous circumference within the target range of contraction.

Each trial of the task consisted of 10 consecutive contraction (1.55 s) and relaxation (0.55 s) periods (total, 21 s) with one hand (Figure 1A). The dynamic grip was executed with the hand indicated at the beginning of the trial by synthetic voice 2.5 s before the “Go” signal (used as the origin of time, *t* = 0 s, for each trial). All participants did a balanced amount of left- and right-hand trials of at least 10 (max. 13) trials per hand. We limited the number of trials to avoid effects of muscular fatigue given the relatively high contractions that the task demanded. Left and right-hand conditions were pseudorandomized across trials to avoid anticipation and interference between conditions. The refreshing rate of the visual feedback overlaid with the force produced in real-time. Each trial was followed by a randomized resting period uniformly distributed between 15 and 21 s, to avoid phasic constructive interference of systemic artifacts, e.g., Mayer waves, in the brain responses.

## Data Collection Pipeline and Methods

**Figure 2:**
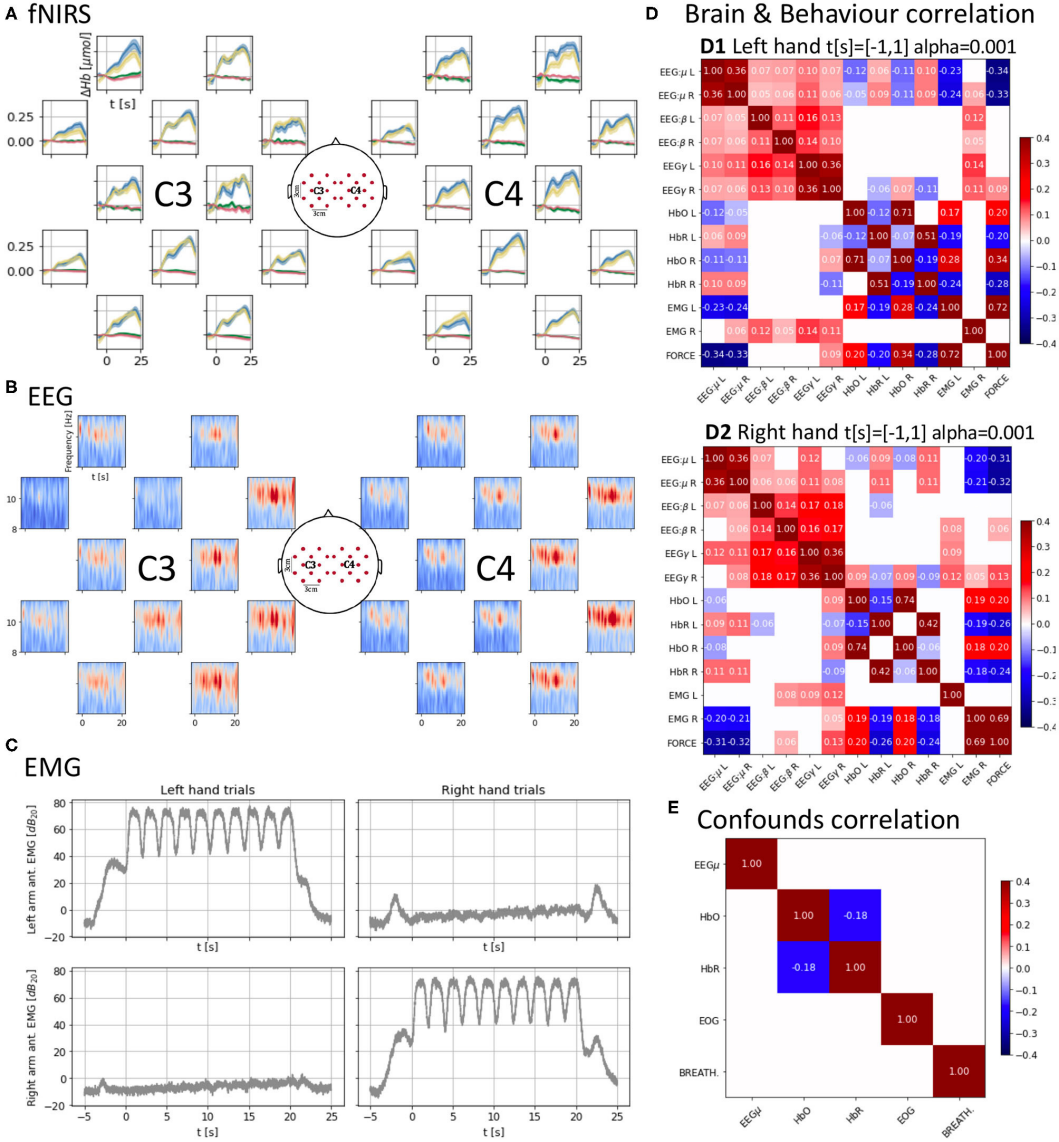
**(A)** Cross-subject average of HbO and HbR changes across trials, time-locked to the “Go” cue (*t* = 0 s) of each trial. Each trial consists of 10 contraction/relaxation periods (21 s of activity starting at the “Go” cue). HbO increases during the task and HbR decreases at a smaller scale, both start returning to baseline after the 21 s of activity. Units in top-left. **(B)** Cross-subject mu-band spectrogram averaged across right-hand trials as in **(B)**. Periodic desynchronizations can be observed. Units in top-left. **(C)** Averaged EMG spectral density across trials as in **(A,B)** showing similar power density for the active muscles controlling the hands. **(D)** Brain and behavior correlation matrix computed on the force onset (−1 s to 1 s around the “Go” cue) showing only significant (*p* < 0.001) correlations. It shows a contralateral change in correlation values between HbO and HbR and force and the time-locked mu desynchronization event represented as the negative correlation between force and the EEG mu power band. **(E)** Confounds correlation matrix showing only significant (*p* < 0.001) correlations computed along the task (*t* = [−1, 20] s around the “Go” cue) for the right hand condition. Correlations between brain signals and confounds are non-significant (α = 0.001).

We recorded multiple signals representing brain activity, motor behavior, and confounds (Figures 1C, 2A–C). The signals capturing brain activity consisted of electroencephalography (EEG) and functional near-infrared spectroscopy (fNIRS). Motor behavior was captured by the force sensor on which the subjects gripped and surface electromyography on both forearms. Potential confounds interfering (breathing and EOG) with EEG and fNIRS were also recorded. A total of three recording devices were used to record all the signals. fNIRS was recorded using a NIRScout system (NIRx Medizintechnik GmbH, Berlin, Germany). EEG, EMG, and EOG were recorded together with an ActiChamp amplifier (BrainProducts, Berlin, Germany). Force and breathing were recorded with a PowerLab 4/25T system (ADInstruments, Castle Hill, Australia). To synchronize the devices, the same computer used to present the task and visual feedback was used to send time-stamping signals to the three devices simultaneously at the beginning and end of the recording and every “Go” cue and were stored by each device in its time reference. The timestamps are used to locate the positions of the same event across different devices and align the measures to the events shown in the computer used to present the task. The sampling frequencies (12.5 Hz for fNIRS and 4 kHz for remaining measures) were selected so that they had a common divisor facilitating the resampling processes without the need to round up due to inexact divisors.

### Brain Signals

All brain signals were non-invasively recorded. A custom 3D printed (formlabs Form2, Formlabs Inc., Somerville, MA 02143, USA) holder made of flexible resin (formlabs RS-F2-FLGR-02) was used to align the fNIRS and EEG sensors to approximately target similar cortical areas (Figure 1B). The sensor layout was configured to result in 12 hybrid EEG-fNIRS recording locations per hemisphere. These locations were homogeneously spread with a 3 cm separation creating a grid. Each hemispherical grid was centered around the corresponding 10-20 system C3 and C4 location.

fNIRS signals were recorded using a NIRScout system (NIRx Medizintechnik GmbH, Berlin, Germany). We used a total of 12 optodes per hemisphere (10 sources and eight detectors in total) sampling at 12.5 Hz. An optode is a source-detector pair 3 cm apart from each other (allowing light to reach an approximate 1.5 cm depth into the skull). fNIRS sources and sensors were laid out to result in 12 optodes. The sources and sensors were symmetrically laid around C3 and C4 positions according to the International 10-20 system leaving an inter-optode distance of 3 cm (Figure 1B). Two wavelengths (*wl*_1_ = 760 nm, *wl*_2_ = 850 nm) continuous functional near-infrared spectroscopy (fNIRS) was used to obtain optical absorption densities that were transformed to oxy-hemoglobin [HbO] and deoxy-hemoglobin concentrations [HbR] using the modified Beer-Lambert Law (Cope et al., 1988). The raw optical densities are also provided in the data set.

An ActiChamp amplifier (BrainProducts, Berlin, Germany) operating at 4 kHz (running software BrainVision, v1.20.0801) was used to record EEG. Twelve EEG sensors per hemisphere were placed in between each sensor-detector fNIRS pair overlaying the region measured by that optode (Figure 1B). The reference in our setup corresponds to the standard Cz 10-20 position (Nomenclature, 1991; Klem et al., 1999). The signals were down-pass filtered and downsampled to 1 kHz in the data set.

To enable EEG and fNIRS sensors to record cortical activity from the same cortical locations, we used a non-standard sensor arrangement covering the bilateral motor cortex (Figure 1B). We used a custom sensor holder 3D printed in flexible resin that for every recording channel allowed the EEG sensor to sit on top of the cortical area targeted by a corresponding fNIRS source-detector pair. Namely, for every fNIRS source-detector pair, an EEG electrode was placed in between. Each holder consisted of a 5 × 5 grid of circular holes whose centers were 1.5 cm apart allowing the required 3 cm separation between fNIRS source-detector pairs with an EEG sensor occupying a hole in between. The positions in the 5 × 5 grid marked in red in Figure 1B correspond to the physical location of EEG electrodes and the approximate recording areas of EEG electrodes and fNIRS source-detector pairs. Physical locations of fNIRS sources and detectors are marked, respectively by “S” and “D” in Figure 1B. A total of 12 recording sites were used per hemisphere due to the limitation of space to fit the multi-modal sensors together. A holder was placed on each hemisphere and held in position using elastics. The disposition of every sensing point is symmetrical to the scalp mid-line, and each grid is centered in the corresponding C3 or C4 site depending on the side so that the central hole overlaid the respective central position. C3 and C4 were located per subject following the 10-20 standard (Nomenclature, 1991; Klem et al., 1999), i.e., at a 20% of the distance between the pre-auricular points passing over the top of the head from the mid-line. The spherical coordinates of the standard positions are provided in the data set.

### Motor Signals

We recorded the grip force and muscular electrical activity to represent motor behavior during the task.

Bilateral bipolar surface electromyography (EMG) was recorded over the longitudinal axis (+ distal and − proximal) of the muscle belly of the *flexor digitorum superficialis* (4 kHz, on the Aux channels of the BrainVision ActiChamp) placed in the anterior and posterior forearm faces (Figure 1B). Before electrode placement, the skin was cleaned with abrasive pads and alcohol to eliminate dead skin cells and fat impact on electrical recording quality. EMG signals were down-pass filtered and downsampled to 1 kHz in the data set.

The dynamic gripping task was conducted using a continuously recorded grip force transducer (PowerLab 4/25T, ADInstruments, Castle Hill, Australia) sampling at 1 kHz. The signal was also used to provide real-time visual display feedback to the subject and the target force level they were asked to produce (Figure 1A). The Maximum Voluntary Contraction (MVC) force for each subject's hand was measured and computed using the same transducer. The recorded force signals, in Volts, were down-pass filtered and downsampled to 50 Hz in the published data set.

### Recording of Potential Confounds (EOG and Breathing)

To complete the picture provided by the data, we recorded potential confounds in the brain and motor behavior signals of interest. The pulse and breathing rate have an impact at the body level on the concentration of hemoglobin and therefore can have an impact on brain and scalp levels of hemoglobin concentration. Whereas pulse is easily removed in the fNIRS analysis band (0.01–0.25 Hz), the breathing rate can overlap with it (Pinti et al., 2019). Sources of muscular electrical activity can spread to the EEG sensors and include eye muscles and skeletal muscles. Thus, we consider electrooculography (EOG), which also carries information on blinks, and breathing as pure confounds. However, EMG might also leak into the EEG sensors and can carry confound information at the same time it provides behavioral information.

#### Electrooculography (EOG)

Bipolar EOG was recorded on the vertical axis (top +, bottom −) of the right eye for all subjects (4 kHz, BrainVision ActiChamp, BrainProducts GmbH, Germany). The signal was down-pass filtered and downsampled to 1 kHz before being included in the data set. We note that the EOG of participant “I” is absent. Nonetheless, this participant was included as the impact of the EOG in the recording locations can be less severe than for frontal recording sites can be corrected using techniques like Independent Component Analysis (ICA) (Onton and Makeig, 2006).

#### Breathing

We captured breathing as the chest diametrical changes during inspiration and expiration. A variable resistor placed inside an elastic strap adjusted around the chest at the level of the Xiphoid process (Figure 1B) was used to record the expansion and contraction of the thoracic cage (1 kHz, PowerLab 4/25T, ADInstruments, Castle Hill, Australia). The signal was down-pass filtered and downsampled to 50 Hz before being included in the data set.

## Data Set Overview

The data set, provided as a single hard-disk file (HDF), has undergone very little processing to avoid biasing future analyses. Here, we make the raw data available and provide companion code that preprocesses the raw data into a readily usable data set (Data Readiness Level C). Preprocessing comprises down-sampling to reduce storage space and the formatting of data, recorded events, and other meta-data from different devices so that all data followed the same format regardless of their device origin. Further preprocessing can be directly applied using the utils python package provided, making the data readily available to exploit in popular python machine learning packages as pytorch and tensorflow.

### Companion Code

The utils package only depends on the public python packages, h5py, numpy, scipy, scikitlearn, matplotlib, which need to be installed. The notebook presentation.ipynb contains a thorough explanation and examples of how the tools in utils can be used to process the data and depends on jupyter. A conda environment.yml file is provided with all dependencies to facilitate installation. All together make the data readily available to exploit, i.e., Data Readiness Level C (Lawrence, 2017).

### Data Set Organization

The data set file structure follows a tree-like organization in three levels. In the first and third levels, the data set contains meta-data in *string* format that can be accessed via the attributes of the level. The first level is the data set itself and the shared attributes across subjects measures, e.g., sampling frequencies and units, and other information such as the channel grid disposition and a template of hybrid sensor spherical coordinates over the scalp. In the second level, the data set is organized in one group per subject indexed by their anonymized ID (i.e., 14 groups with keys A to N) and contain no attributes. In the third level, each group contains a subgroup for each measure (e.g., keys frc for force and eeg for EEG) containing the data in *numeric* format and an attribute called events containing the times at which a timestamp was received during the recording (e.g., “relax,” “left-hand,” “right-hand”) and the “begin” and “end” timestamps indicating the beginning and end of the recording session, also *numeric*. In particular, the events group contains a second *numeric* attribute MVC containing the maximum voluntary contraction value for each hand.

### Data Validation Preprocessing Pipeline

The following preprocessing was applied to the raw signals in the data set to obtain the brief analysis in Figure 2, which can be reproduced using the companion notebook. After preprocessing all signals, epochs were extracted from 5 s before the “Go” instruction to 25 s after.

#### fNIRS

The optical intensity, ${\hat{\text{I}}}_{ij}^{\lambda }$, for each wavelength, λ, was low-pass filtered below 0.25 Hz with a 7th order elliptical filter. Changes in optical densities per wavelength, $\Delta {\text{OD}}_{ij}^{\lambda }\left(t\right)$, were obtained using

(1) $$\begin{array}{l} \Delta {\text{OD}}_{ij}^{\lambda }\left(t\right)=-\text{log}\left({\hat{\text{I}}}_{ij}^{\lambda }\left(t\right)\;/\;{\bar{\text{I}}}_{ij}^{\lambda }\right) \end{array}$$

with *i* and *j* the indices of valid sensor-detector pairs respectively, *t* the time and ${\bar{\text{I}}}_{ij}^{\lambda }$ the average of the optical intensity 1 s prior to the “Go” instruction. Oxygenated and deoxygenated hemoglobin concentration changes, ΔHbO and ΔHbR, respectively, were computed solving the modified Beer-Lambert law (Cope et al., 1988),

(2) $$\begin{array}{l} \Delta {\text{OD}}_{ij}^{\lambda }={\text{L}}_{ij}^{\lambda }{\text{DPF}}^{\lambda }\left(\epsilon_{\text{HbR}}^{\lambda }\Delta \text{HbR}+\epsilon_{\text{HbO}}^{\lambda }\Delta \text{HbO}\right) \end{array}$$

with DPF^λ^, the dimensionless *differential path-length factor* accounting for the reduction in intensity due to scattering tissues (DPF^760^ = 5.98 and DPF^850^ = 7.54); $\epsilon_{\text{Hb}}^{\lambda }$, the *molar extinction coefficient* for each hemoglobin and wavelength in mol^−1^cm^−1^ accounting for the absorption of light ($\epsilon_{\text{HbO}}^{760}=1486.6$, $\epsilon_{\text{HbR}}^{760}=3843.7$, $\epsilon_{\text{HbO}}^{850}=2526.4$, and $\epsilon_{\text{HbR}}^{850}=1798.6$); and L the source-detector distance in cm (L = 3cm). After this preprocessing, the average and the standard error of the mean across subjects for each hand condition and hemoglobin type were plotted in the corresponding position of the 2D layout (Figure 2A). We can observe an increase and decrease of HbO with the task onset (*t* = 0 s) and end (*t* = 21 s), respectively. We can also identify several peaks in the average response which might be a result of the on-off dynamics of the task which might introduce small variations on the global trend of Hb variations. Opposite changes can be observed for HbR at a smaller scale.

#### EEG

EEG was first downsampled to 250 Hz (with anti-aliasing down-pass filtering). Notch filters were applied at the mains (50 Hz) and fNIRS (12.5 Hz) frequencies and their harmonics. EEG was finally high-pass filtered above 1 Hz using a 5th order Butterworth filter. ICA was used in two stages to remove components correlated first with EOG and second with EMG. ICA related preprocessing only affected the signals used to compute the mixing matrix, which was then applied to the data going through the main EEG pipeline. For EOG, both the EOG and EEG were downsampled to 25 Hz. A maximum of 1 independent component correlated above 0.3 (in absolute values) with the EOG was rejected. For EMG, both the EMG and EEG were downsampled to 125 Hz. The rejection of components was stricter to ensure EMG was not contaminating the data. One component was rejected whenever its correlation magnitude with any of the recorded EMG channels was >10^−4^. Figure 2B presents the averaged spectrogram across subjects for the right-hand condition (the left-hand condition can be found in the notebook) for the mu band (8–13 Hz). Interestingly, the on-off nature of the task might be introducing periodic variations of power in the mu band due to desynchronization (Pfurtscheller et al., 2006).

#### EMG

The EMG was also first downsampled to 250 Hz (with anti-aliasing down-pass filtering), and it was then high-pass filtered with a 17th order Butterworth filter of above 110 Hz. To generate Figure 2C we computed the Hilbert envelope of the signal and used it to obtain decibels of power density referred to the mean power of the signal during the epoch. We finally averaged these power densities across subjects per hand condition and arm location of the electrodes. The active EMG (i.e., those corresponding to the arm used during each hand condition) carry a similar amount of power density for each hand condition. The passive electrodes have a much flatter amount of power density during the task and instead have clear peaks at the beginning and end of the trials when the subjects were allowed to relax.

#### Force

The force signal was band-pass filtered between 10^−4^ and 9 Hz (second order elliptical filter). Once epochs were extracted, it was again high-pass filtered above 10^−3^ Hz to remove any remaining offset. These low high-pass frequencies were selected to preserve the squared shape of the forces, which are very rich in low frequencies. Once the offset was removed, voluntary contraction values were obtained by dividing the resulting forces by the maximum voluntary contraction force recorded at the beginning of the trial. Figure 1A shows the gathered trials for all subjects per hand condition. Subjects mostly engaged with the task in timing and contraction values with the left condition presenting slightly more overshoots. Although the task is conceptually simple, the provision of only partial visual feedback and its fast on-off nature contributed to higher variability in the behavior within the desired levels of contraction. We consider these aspects to be more representative of natural force applications where feedback is more proprioceptive and changes in force can be fast and span a wider range than discrete target levels.

#### Other Signals (EOG and Breathing)

EOG was downsampled (with prior anti-aliasing filtering) to 50 Hz. Then filtered using an 8th order high-pass filter above 1 Hz. Breathing was also low-pass filtered with a 6th order Butterworth filter below 0.25 Hz.

### Correlations Across Multi-Modal Signals and Validation

After preprocessing, signals were correlated to characterize a “brain and behavior” or neurobehavioral correlation (EEG, fNIRS, EMG, and force in Figure 2D) and a “confounds” correlation structure (EEG, fNIRS, EOG, and breathing in Figure 2E). EEG and EMG underwent additional filtering and spectral density computation similarly to that used for the EMG plots. For EEG the mu (8–12 Hz), beta (12–30 Hz), and gamma (60–125 Hz) bands spectral densities were extracted. Then, signals were downsampled to the lowest sampling frequency in the data set, i.e., 12.5 Hz, and cropped between −1 and 1 s (“Go” cue at *t* = 0 s) to focus on the onset of motor activity in the case of the “brain and behavior” correlation and between −1 and 21 s for the “confounds.” The signals were finally normalized. For each hand condition, the “brain and behavior” correlation (Figure 2D) was computed over the appended observations corresponding to the *t* = [−1, 1] s crops for all combinations of the left and right hemisphere mu, beta, and gamma EEG power bands and HbO and HbR, the right- and left-arm EMG and force. For the brain signals, channels 4 and 16 (Figure 1B) were selected as representative of the corresponding hemisphere activity. A similar process was used for the “confounds” correlation for the *t* = [−1, 20] s. A significance level α = 0.001 is set and only significant correlation values are shown.

There is a very small correlation between brain signals and confounds (<0.03 in absolute values, not shown due to lack of significance α = 0.001, Figure 2E). This suggests that the confounds do not interfere with the recorded signals after applying the standard preprocessing pipeline to the raw data.

The key observations of our data can be found for the “brain and behavior” correlation (Figure 2D). For considering the interaction between brain signals (fNIRS and EEG) and task measure (force) we need to bear in mind that the time scales of fNIRS and EEG are very different (seconds vs. milliseconds). In the EEG domain, it is known that motor activity onsets are reflected in EEG power features. In our data, we observe significant strong anti-correlation (*r* ≈ −0.3, *p* < 0.001) between the EEG power in the mu band and the force which indicates that passing from a resting state (high mu power) to motor activity (low mu power) is properly captured by these spectral features when we look at a time window from −1 to 1 s around the Go cue. This decrease in power, also known as *mu event-related desynchronization* (mu-ERD), is due to the desynchronization of neuronal activity (Pfurtscheller et al., 2006), and it shows that the EEG is aligned with the force, helping to further confirm the validity of our data set.

However, when the correlation is computed focusing on the 20 s of the task (from 0 s before the Go cue to 20 s after), the EEG mu-ERD is not significant (*p*> 0.001). Only the beta band (12–30 Hz), known to be synchronized with motor activity (Kristeva-Feige et al., 2002), appears with a low level positive correlation (*r* ≈ 0.04−0.05, *p* < 0.001). The lack of mu-ERD and force correlation during the 20 s of continuous contraction/relaxations might be a consequence of the velocity at which the sequential contractions/relaxations were executed, not leaving enough time to the motor cortex to reach a synchronized equilibrium state before it was desynchronized again. Furthermore, this can also indicate that mu frequencies (8–12 Hz) are not fast enough to track this kind of subtle phase changes and a justification to develop more precise algorithms or feature extractors as suggested by Paek et al. (2019).

In the fNIRS domain ±1 s around the “Go” cue, we also observe a typical HbO/HbR anticorrelation (*r* ≈ 0.15, *p* < 0.001) (Jasdzewski et al., 2003; Huppert et al., 2006) in the structure with higher magnitudes present for the contralateral hemisphere to the hand used. The HbO/HbR anticorrelation is stronger in the right hemisphere for the left hand although also present in the left hemisphere, and it is stronger in the right hemisphere. This suggests that the dominant right hand may engage the left hemisphere while the non-dominant left hand engages both hemispheres with a preference for the contralateral one. HbO and HbR also show significant correlation with the force (*r* ≈ 0.2−0.3, *p* < 0.001). In particular, HbR, which is more specific than HbO (Hirth et al., 1997), also shows higher anticorrelation with the force for the contralateral hemisphere.

Finally, there is a strong significant correlation (*r* ≈ 0.7, *p* < 0.001) between the EMG power envelopes of the active hands and the force which supports the synchronization of the different devices used to record these measures.

This brief analysis aims to validate the data set and present some of its features. We encourage the community to develop algorithms to better understand the rich temporal relationships between brain signals spanning very different time scales and physical origins present in the data set with BCI and neuroimaging purposes.

## Data Availability Statement

The HYGRIP data set can be downloaded from https://doi.org/10.6084/m9.figshare.12383639.v1 and the companion code can be cloned from https://gitlab.doc.ic.ac.uk/bbl/hygrip.git.

## Ethics Statement

The studies involving human participants were reviewed and approved by Imperial College London Research Ethics Committee. The patients/participants provided their written informed consent to participate in this study.

## Author Contributions

AF conceived the project. PO and AF designed the experiment, analyzed the data, and edited the manuscript. PO and TZ performed the data recordings. PO wrote the companion code and drafted the paper. All authors contributed to the article and approved the submitted version.

## Conflict of Interest

The authors declare that the research was conducted in the absence of any commercial or financial relationships that could be construed as a potential conflict of interest.

## Footnotes

^1^Raw data (2.8 h of data- worth time, 328 trials and 14 subjects) available at https://doi.org/10.6084/m9.figshare.12383639.v1.

^2^Companion code available at: https://gitlab.doc.ic.ac.uk/bbl/hygrip.git.

## References

[B1] Ahn S. Jun S. C. (2017). Multi-modal integration of EEG-fNIRS for brain-computer interfaces-current limitations and future directions. Front. Hum. Neurosci. 11:503. 10.3389/fnhum.2017.00503 29093673 PMC5651279

[B2] Anumanchipalli G. K. Chartier J. Chang E. F. (2019). Speech synthesis from neural decoding of spoken sentences. Nature 568, 493–498. 10.1038/s41586-019-1119-1 31019317 PMC9714519

[B3] Belić J. J. Faisal A. A. (2015). Decoding of human hand actions to handle missing limbs in neuroprosthetics. Front. Comput. Neurosci. 9:27 10.3389/fncom.2015.00027 25767447 PMC4341559

[B4] Bocquelet F. Hueber T. Girin L. Savariaux C. Yvert B. (2016). Real-time control of an articulatory-based speech synthesizer for brain computer interfaces. PLoS Comput. Biol. 12:e1005119 10.1371/journal.pcbi.1005119 27880768 PMC5120792

[B5] Cope M. Delpy D. Reynolds E. Wray S. Wyatt J. Van der Zee P. (1988). Methods of quantitating cerebral near infrared spectroscopy data, in Oxygen Transport to Tissue X, eds M. Mochizuki, C. R. Honig, T. Koyama, T. K. Goldstick, and D. F. Bruley (New York, NY: Springer), 183–189. 10.1007/978-1-4615-9510-6_21 3129910

[B6] Ganzer P. D. Colachis S. C. IV. Schwemmer M. A. Friedenberg D. A. Dunlap C. F. Swiftney C. E. (2020). Restoring the sense of touch using a sensorimotor demultiplexing neural interface. Cell 181, 763–773.e12. 10.1016/j.cell.2020.03.054 32330415

[B7] Guenther F. H. Brumberg J. S. Wright E. J. Nieto-Castanon A. Tourville J. A. Panko M.. (2009). A wireless brain-machine interface for real-time speech synthesis. PLoS ONE 4:e8218. 10.1371/journal.pone.0008218 20011034 PMC2784218

[B8] Hirth C. Obrig H. Valdueza J. Dirnagl U. Villringer A. (1997). Simultaneous assessment of cerebral oxygenation and hemodynamics during a motor task, in Oxygen Transport to Tissue XVIII, eds E. M. Nemoto and C. J. LaManna (New York, NY: Springer), 461–469. 10.1007/978-1-4615-5865-1_59 9269463

[B9] Hochberg L. R. Bacher D. Jarosiewicz B. Masse N. Y. Simeral J. D. Vogel J. (2012). Reach and grasp by people with tetraplegia using a neurally controlled robotic arm. Nature 485, 372–375. 10.1038/nature11076 22596161 PMC3640850

[B10] Huppert T. J. Hoge R. D. Diamond S. G. Franceschini M. A. Boas D. A. (2006). A temporal comparison of BOLD, ASL, and NIRS hemodynamic responses to motor stimuli in adult humans. NeuroImage 29, 368–382. 10.1016/j.neuroimage.2005.08.065 16303317 PMC2692693

[B11] Jasdzewski G. Strangman G. Wagner J. Kwong K. Poldrack R. Boas D. (2003). Differences in the hemodynamic response to event-related motor and visual paradigms as measured by near-infrared spectroscopy. Neuroimage 20, 479–488. 10.1016/S1053-8119(03)00311-2 14527608

[B12] Jochumsen M. Niazi I. K. Mrachacz-Kersting N. Farina D. Dremstrup K. (2013). Detection and classification of movement-related cortical potentials associated with task force and speed. J. Neural Eng. 10:056015. 10.1088/1741-2560/10/5/056015 23986024

[B13] Klem G. H. Lüders H. O. Jasper H. Elger C. (1999). The ten-twenty electrode system of the international federation. Electroencephalogr. Clin. Neurophysiol. 52, 3–6. 10590970

[B14] Kristeva-Feige R. Fritsch C. Timmer J. Lücking C.-H. (2002). Effects of attention and precision of exerted force on beta range EEG-EMG synchronization during a maintained motor contraction task. Clin. Neurophysiol. 113, 124–131. 10.1016/S1388-2457(01)00722-2 11801434

[B15] Lawrence N. D. (2017). Data readiness levels. arXiv [preprint] arXiv:1705.02245.

[B16] Meng J. Zhang S. Bekyo A. Olsoe J. Baxter B. He B. (2016). Noninvasive electroencephalogram based control of a robotic arm for reach and grasp tasks. Sci. Rep. 6:38565. 10.1038/srep38565 27966546 PMC5155290

[B17] Nomenclature S. E. P. (1991). American electroencephalographic society guidelines for. J. Clin. Neurophysiol. 8, 200–202. 10.1097/00004691-199104000-00007 2050819

[B18] Oldfield R. C. (1971). The assessment and analysis of handedness: the Edinburgh inventory. Neuropsychologia 9, 97–113. 10.1016/0028-3932(71)90067-4 5146491

[B19] Onton J. Makeig S. (2006). Information-based modeling of event-related brain dynamics. Prog. Brain Res. 159, 99–120. 10.1016/S0079-6123(06)59007-7 17071226

[B20] Ortega P. Colas C. Faisal A. A. (2018). Compact convolutional neural networks for multi-class, personalised, closed-loop EEG-BCI, in 2018 7th IEEE International Conference on Biomedical Robotics and Biomechatronics (Biorob) (Enschede), 136–141. 10.1109/BIOROB.2018.8487644

[B21] Ostry D. J. Feldman A. G. (2003). A critical evaluation of the force control hypothesis in motor control. Exp. Brain Res. 153, 275–288. 10.1007/s00221-003-1624-0 14610628

[B22] Paek A. Y. Gailey A. Parikh P. J. Santello M. Contreras-Vidal J. L. (2019). Regression-based reconstruction of human grip force trajectories with noninvasive scalp electroencephalography. J. Neural Eng. 16:066030 10.1088/1741-2552/ab4063 31476751

[B23] Pfurtscheller G. Brunner C. Schlögl A. Da Silva F. L. (2006). Mu rhythm (de) synchronization and EEG single-trial classification of different motor imagery tasks. NeuroImage 31, 153–159. 10.1016/j.neuroimage.2005.12.003 16443377

[B24] Pfurtscheller G. Müller G. R. Pfurtscheller J. Gerner H. J. Rupp R. (2003). Thought-control of functional electrical stimulation to restore hand grasp in a patient with tetraplegia. Neurosci. Lett. 351, 33–36. 10.1016/S0304-3940(03)00947-9 14550907

[B25] Pinti P. Scholkmann F. Hamilton A. Burgess P. Tachtsidis I. (2019). Current status and issues regarding pre-processing of fNIRS neuroimaging data: an investigation of diverse signal filtering methods within a general linear model framework. Front. Hum. Neurosci. 12:505 10.3389/fnhum.2018.00505 30687038 PMC6336925

[B26] Royer A. S. Doud A. J. Rose M. L. He B. (2010). EEG control of a virtual helicopter in 3-dimensional space using intelligent control strategies. IEEE Trans. Neural Syst. Rehabil. Eng. 18, 581–589. 10.1109/TNSRE.2010.2077654 20876032 PMC3037732

[B27] Thomik A. A. Haber D. Faisal A. A. (2013). Real-time movement prediction for improved control of neuroprosthetic devices, in 2013 6th International IEEE/EMBS Conference on Neural Engineering (NER) (San Diego, CA), 625–628. 10.1109/NER.2013.6696012

[B28] Vidal J. J. (1973). Toward direct brain-computer communication. Annu. Rev. Biophys. Bioeng. 2, 157–180. 10.1146/annurev.bb.02.060173.001105 4583653

[B29] Vidal J. J. (1977). Real-time detection of brain events in EEG. Proc. IEEE 65, 633–641. 10.1109/PROC.1977.10542

[B30] Walker I. Deisenroth M. Faisal A. (2015). Deep Convolutional Neural Networks for Brain Computer Interface Using Motor Imagery. Imperial College of Science, Technology and Medicine, Department of Computing, 68.

[B31] Wang K. Wang Z. Guo Y. He F. Qi H. Xu M. (2017). A brain-computer interface driven by imagining different force loads on a single hand: an online feasibility study. J. Neuroeng. Rehabil. 14:93 10.1186/s12984-017-0307-1 28893295 PMC5594542

[B32] Westling G. Johansson R. (1984). Factors influencing the force control during precision grip. Exp. Brain Res. 53, 277–284. 10.1007/BF00238156 6705863

[B33] Wolpaw J. R. Birbaumer N. Heetderks W. J. McFarland D. J. Peckham P. H. Schalk G. (2000). Brain-computer interface technology: a review of the first international meeting. IEEE Trans. Rehabil. Eng. 8, 164–173. 10.1109/TRE.2000.847807 10896178

[B34] Wolpaw J. R. Birbaumer N. McFarland D. J. Pfurtscheller G. Vaughan T. M. (2002). Brain-computer interfaces for communication and control. Clin. Neurophysiol. 113, 767–791. 10.1016/S1388-2457(02)00057-3 12048038

[B35] Wolpaw J. R. McFarland D. J. (2004). Control of a two-dimensional movement signal by a noninvasive brain-computer interface in humans. Proc. Natl. Acad. Sci. U.S.A. 101, 17849–17854. 10.1073/pnas.0403504101 15585584 PMC535103

[B36] Xiloyannis M. Gavriel C. Thomik A. A. Faisal A. A. (2017). Gaussian process autoregression for simultaneous proportional multi-modal prosthetic control with natural hand kinematics. IEEE Trans. Neural Syst. Rehabil. Eng. 25, 1785–1801. 10.1109/TNSRE.2017.2699598 28880183

[B37] Yin X. Xu B. Jiang C. Fu Y. Wang Z. Li H.. (2015). A hybrid BCI based on EEG and fNIRS signals improves the performance of decoding motor imagery of both force and speed of hand clenching. J. Neural Eng. 12:036004. 10.1088/1741-2560/12/3/036004 25834118

